# Well-differentiated HPV-independent gastric-type adenocarcinoma of the cervix: a case report and literature review

**DOI:** 10.3389/fonc.2025.1617050

**Published:** 2025-11-24

**Authors:** Weijun Wang, Yuan Cai, Zhuanqin Ren, Hongzhe Tian, Xia Liu

**Affiliations:** 1 Medical Imaging Department of Baoji Central Hospital, Baoji, China; 2 Pathology Department of Baoji Central Hospital, Baoji, China; 3 Department of Ultrasound Medicine, Baoji Hospital Affiliated to Xi’an Medical College, Baoji, China

**Keywords:** HPV-independent cervical gastric adenocarcinoma, cervical minimal deviation adenocarcinoma, cervical gastric adenocarcinoma, cervical mucinous adenocarcinoma, HPV-independent

## Abstract

**Background:**

Well-differentiated HPV-independent gastric-type endocervical adenocarcinoma (formerly termed minimal deviation adenocarcinoma) is a rare, aggressive cervical cancer that is challenging to diagnose because its histologic features can be very similar to benign endocervical glands or even normal glands. The imaging features and clinical manifestations are also non-specific, with the disease often having an insidious onset. However, this tumor exhibits a high degree of aggressiveness and tends to progress rapidly; as a result, it is frequently diagnosed at an advanced stage, missing the optimal window for treatment. Therefore, thorough investigations are required to identify specific pathological features unique to this condition to improve imaging and the early diagnosis rate.

**Case introduction:**

This article reports a case of a 42-year-old female patient who was hospitalized for 2 months due to vaginal discharge and 1 week of lower abdominal pain. Upon physical examination, a small amount of odorless, transparent secretion was detected in the vagina; the cervix appeared enlarged, and a mass measuring approximately 6 cm was palpable in the right adnexal region. The pelvic MRI indicated the presence of malignant cervical lesions, potentially cervical gastric-type adenocarcinoma, accompanied by significant pericervical invasion. Colposcopy also suggested the presence of cervical adenocarcinoma. Following the exclusion of any surgical contraindications, robotic-assisted laparoscopic extensive hysterectomy and bilateral adnexectomy were performed under general anesthesia. Postoperative pathological examination confirmed the presence of invasive cervical adenocarcinoma, independent of HPV. The subtype identified was classified as gastric-type adenocarcinoma, exhibiting locally well-differentiated characteristics.

**Conclusion:**

Highly differentiated HPV-independent cervical gastric-type adenocarcinoma is insidious in onset and progresses rapidly. Clinically, it often presents with abnormal vaginal discharge or bleeding, and deep biopsy or cervical conization is often required for diagnosis. Pathologically, it is characterized by randomly distributed, highly differentiated tumoral glands, which often invade deep into the uterine wall, extending beyond 2/3 of the cervical wall. It also shows positive expression of gastric-type markers such as MUC6, Mucin 5AC, and HIK1083. Imaging examination revealed the size of the lesion, depth of infiltration, the invasion of adjacent organs, and lymph node metastasis, which is beneficial for differential diagnosis, clinical staging, and the development of a treatment plan.

## 1 Introduction

Cervical cancer (CC) is the fourth most common cancer in women, and continues to be a serious global health threat, thus necessitating significant intervention. While the primary etiology of cervical cancer is persistent infection with high-risk human papillomavirus (HPV), a small percentage of cases are not linked to HPV infection. Expanding HPV vaccinations and screening programs has led to significant progress in preventing and treating HPV-associated cervical cancer (1, 2). Conversely, while the incidence of HPV-related cervical cancers is decreasing, HPV-independent cervical cancers appear to be on the rise. The well-differentiated variant of ​HPV-independent gastric-type endocervical adenocarcinoma (G-EAC), formerly called​ minimal deviation adenocarcinoma of the cervix (MDA), is a special type of cervical mucinous adenocarcinoma (3, 4). It is clinically rare, accounting for approximately 1% of all cervical cancers. The pathological term, minimal deviation, has been used to describe its high degree of differentiation. Due to the high degree of differentiation and minimal cytological atypia, G-EAC is histologically challenging to distinguish from benign endocervical lesions or even normal glands, resulting in a high risk of missed diagnosis or misdiagnosis (5). Its imaging manifestations are non-specific, and the clinical features are atypical, contributing to a high rate of diagnostic error (6). This disease typically has an insidious onset but is highly aggressive and progresses rapidly. Patients are often diagnosed at an advanced stage, missing the optimal window for treatment, which results in a very poor prognosis (7). Therefore, early diagnosis and treatment of this adenocarcinoma continue to pose a significant challenge. This article reports a case of localized, well-differentiated G-EAC that was confirmed by postoperative pathology. Literature related to this disease was examined, and the clinical, imaging, and pathological characteristics were discussed to foster a comprehensive understanding of it pathophysiology and improve its early detection rate.

## 2 Case introduction

A 42-year-old female patient was hospitalized for 2 months due to vaginal discharge and 1 week of lower abdominal pain. She developed vaginal discharge that was clear and varied in quantity, without any odor, 2 months before admittance. She showed improvement but still had some discharge after receiving anti-infective treatment at a local county hospital. She then experienced intermittent, cramping lower abdominal pain 1 week later without any apparent cause, which was not accompanied by diarrhea, abdominal distension, or any other discomfort. This led to her hospitalization in the local county hospital for treatment. Transvaginal ultrasound and abdominal CT scans revealed a cystic and solid mass in the right adnexal region, intrauterine fluid accumulation, and an enlarged cervix. The cervical TCT and HPV test were found to be normal, while vaginal biopsy pathology revealed the presence of parabasal cell hyperplasia in the local area of the cervix. Segmental curettage was then performed to diagnose uterine and cervical endometrial disease. The examination indicated the presence of irregular hyperplastic changes in the endometrium, and the tissue obtained from the cervical canal could not rule out the possibility of cervical gastric-type adenocarcinoma. During the hospitalization, following the administration of anti-infective treatment, the abdominal pain slightly improved compared to before. However, the pain intensified once the medication was stopped. It was recommended to seek treatment at a higher-level hospital, and the patient was subsequently transferred to our hospital. The patient has undergone two cesarean deliveries, resulting in the birth of one son and one daughter, both of whom are healthy. Her menstrual cycle is regular. She had her first menstruation at the age of 15, with a cycle of 30 days and a duration of 5 days. Her last menstrual period was on October 7, 2024. Her past menstrual periods were moderate in volume and normal in color, and were accompanied by dysmenorrhea. She occasionally takes oral analgesics and has no blood clots. She got married at the age of 25, and her spouse is still alive. Both of her parents are alive, and there is no known family history of any genetic diseases. Following a thorough physical examination by a specialist, we noted a small amount of transparent secretion in the vagina, which was odorless; the cervix appeared enlarged, measuring approximately 5 cm in diameter, and showed no bleeding during palpation, exhibiting no tenderness when lifted. A mass measuring around 6 cm in diameter was detected in the right adnexal area, characterized by a smooth surface, limited mobility, and absence of tenderness; no additional abnormalities were identified.

**Figure 1:**
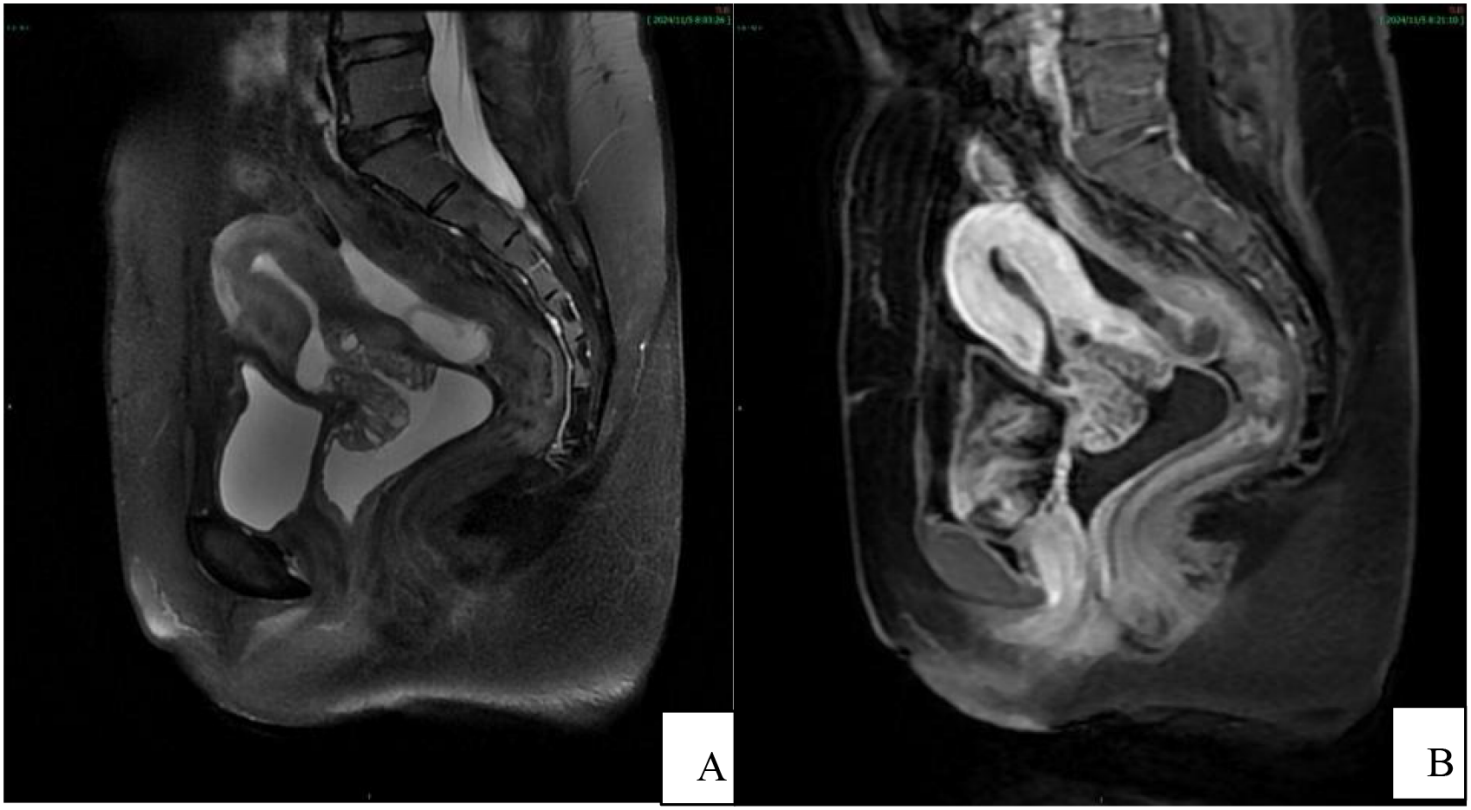
Pelvic MRI sagittal position. **(A)** T2WI shows an enlarged cervix with diffuse, irregularly sized, quasi-circular or small patchy high signal intensity shadows on the cervix wall, with unclear boundaries and some visible fine septa within; the image reveals intrauterine fluid accumulation and post-cesarean section changes on the anterior wall of the uterus; **(B)** In the arterial phase of enhancement, the cervical lesion exhibits uneven enhancement, with significant enhancement of the cyst wall and no obvious enhancement within the cyst. The lesion involves the lower 1/3 of the uterine body and the upper 1/3 of the anterior wall of the vagina, with unclear demarcation between the lesion and the posterior wall of the bladder.

**Figure 2:**
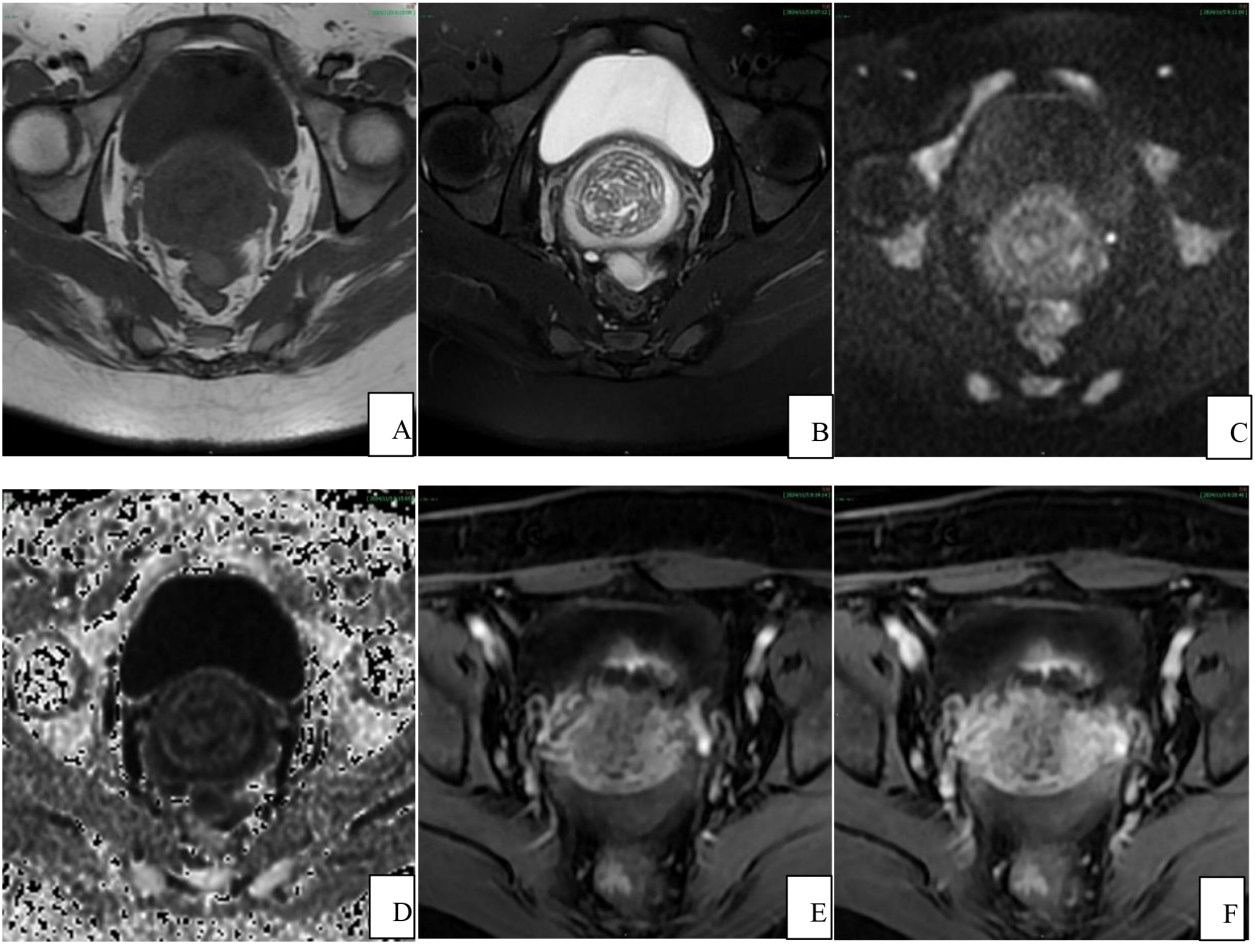
MRI axial scan results of the patient’s pelvic cavity. Diffuse multiple cystic lesions within the enlarged cervical wall, with unclear boundaries and no restricted diffusion. The cyst wall is enhanced on contrast-enhanced scans, while the cyst interior remains unenhanced. The lesions have invaded the subserous layer. **(A)** T1WI; **(B)** T2WI; **(C)** DWI; **(D)** ADC; **(E)** arterial phase after contrast enhancement; **(F)** delayed phase after contrast enhancement.

**Figure 3:**
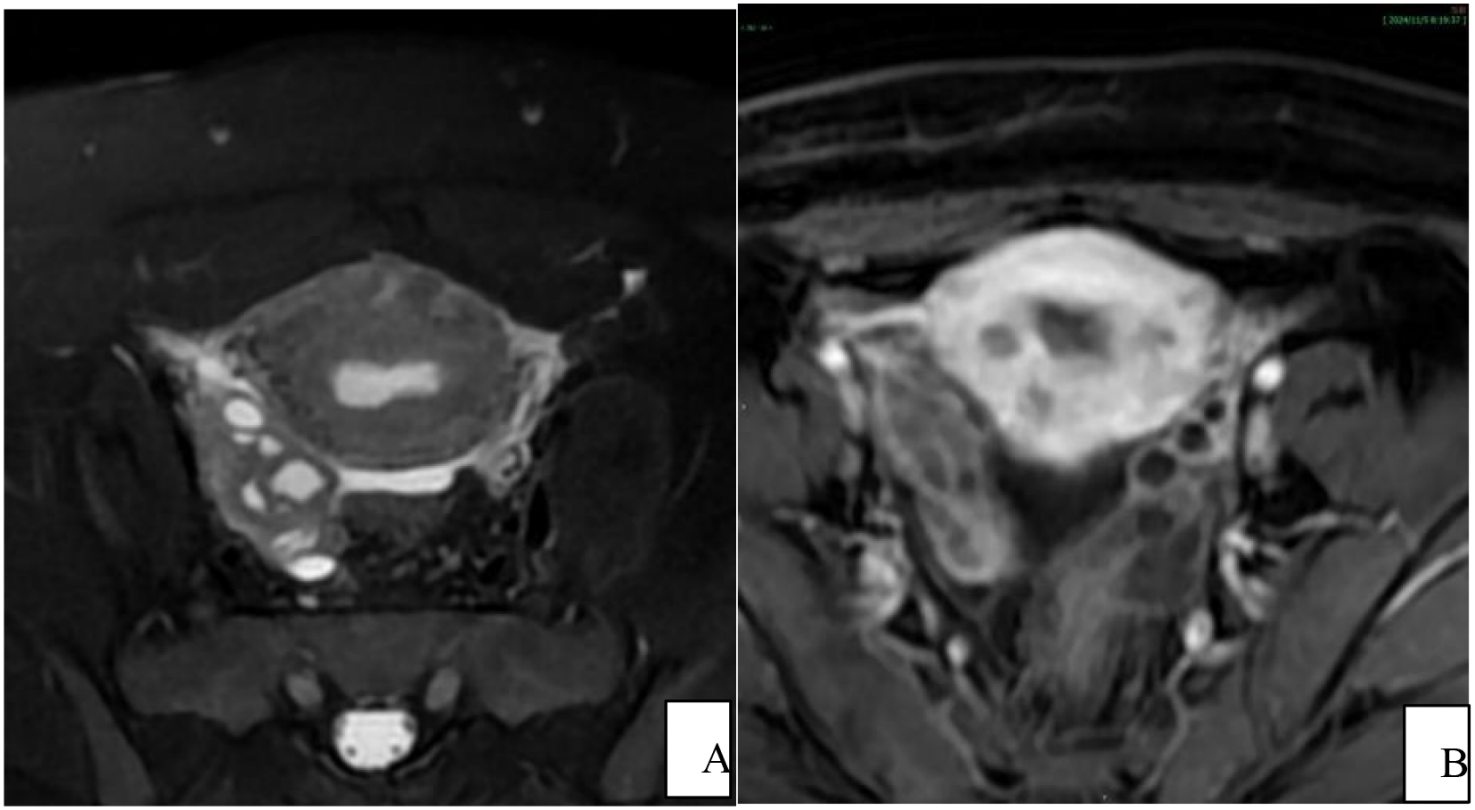
Pelvic MRI axial view. **(A)** T2WI shows uneven signal intensity in the uterine wall; the right ovary is enlarged, with multiple cystic high-signal intensities inside; **(B)** In the arterial phase of enhancement, multiple low-enhancement foci are seen in the uterine wall; multiple cystic shadows with wall enhancement are seen in the right ovary.

The results from the pelvic MRI plain and contrast-enhanced scans (**Figures 1**–**3**) revealed an unremarkable bladder, well-filled with smooth and uniform bladder walls. The uterus showed local thinning of the anterior wall from the cesarean section, and measured approximately 5.6 cm×9.3 cm×6.6 cm. Water-like long T1 and long T2 signal shadows are visible in the uterine cavity, with a normal thickness of the endometrium. The myometrium exhibited an irregular thickness, particularly in the anterior wall, accompanied by uneven signal intensity, and the junction zone remained unclear. The contrast-enhanced scan revealed uneven enhancement of the myometrium, with observable several small, patchy, low-signal non-enhanced shadows. The cervix was enlarged, with an anteroposterior diameter of approximately 3.6 cm. Irregularly shaped diffuse long T1 and long T2 signal shadows were observed in the cervical area, characterized by unclear borders and multiple fine septa. The contrast-enhanced scan demonstrates significant uneven enhancement, with a clear enhancement of the cyst wall measuring approximately 4.6 cm×3.0 cm, and no notable enhancement within the cyst itself. The lesion reached the lower 1/3 of the uterine body and extended down to the upper 1/3 of the anterior vaginal wall, exhibiting unclear demarcation between the lesion and the posterior bladder wall. Water-like long T1 and long T2 signal shadows were observed in the vagina. Additionally, multiple irregularly shaped long or short T1 and long T2 signal shadows were present in both ovarian regions, some of which displayed unclear boundaries and uneven signal intensity, thick and uneven cyst walls. The contrast-enhanced scan showed mild uneven enhancement. The bilateral fallopian tubes were tortuous, dilated, and filled with fluid, with some walls unevenly thickened. The contrast-enhanced scan demonstrated mild enhancement of the tube walls, along with the presence of multiple small nodular protrusions on the walls of the tubes. Water-like long T2 signal shadows were visible in the pelvic cavity. The pelvic wall structure was normal, and no enlarged lymph nodes were observed in the pelvic cavity. Based on the comprehensive results obtained from pelvic MR, malignant cervical lesions were suspected, potentially indicating the presence of cervical gastric-type adenocarcinoma, which exhibited significant invasion surrounding the cervix. Additionally, the possibility of bilateral ovarian and fallopian tube metastasis could not be ruled out.

Results from the transvaginal ultrasound revealed the presence of a mixed echo mass adjacent to the right ovary, along with fluid buildup in the left fallopian tube. These observations are indicative of potential lesions in the fallopian tube, characterized by ambiguous internal dark regions, thickened tube walls, and visible small protrusions. Additionally, there is fluid accumulation within the uterine cavity, accompanied by indistinct dark areas, and cervical hypertrophy featuring multiple retained cysts. A diverticulum was suspected at the site of the cesarean section incision; there were slightly abundant blood flow signals observed at the external cervical orifice. A small amount of fluid accumulation was noted in the pelvic cavity, accompanied by visible fibrous septa and possible pelvic adhesion.

The findings from the laboratory examinations showed that the patient belongs to an Rh^+^ type A blood group. Carbohydrate antigen CA125 was increased, measuring 202.90 U/ml. The blood test for HCG was negative, hence ruling out pregnancy. Plasma D-dimer quantitative detection and coagulation four tests revealed that the levels of fibrinogen and D-dimer were both elevated, measuring 5.17 g/L and 1.67 mg/L, respectively. Blood routine test for CEA, AFP, HE4, SCC, CA199, infectious disease screening, liver and kidney function, electrolyte, blood lipid, and blood glucose were basically normal.

Colposcopy findings showed the presence of cuff-like glandular opening crypts in the cervix; the surface appeared irregular, with atypical vessels observed, while no abnormalities were detected in the vulva and vagina. Cervical adenocarcinoma was suspected.

The morphological analysis and immunohistochemical findings from the pathological evaluation of biopsy specimens from a local hospital corroborated the diagnosis of HPV-independent invasive adenocarcinoma. Immunohistochemical results were as follows: Ki-67 auto (active area 30%), CK7 (+), CK20 (-), CDX2 (-), CEA (+), Mucin 5AC (++), MUC6 (+), P16 (-), PR auto (-).

### 2.1 Diagnosis and differentiation

Clinical diagnosis was as follows: (1) cervical adenocarcinoma; (2) adnexal tumor with possible metastasis; (3) post-cesarean section complications.

The diagnostic basis was as follows: (1) Vaginal discharge for 2 months and lower abdominal pain for 1 week. (2) Gynecological examination revealed cervical enlargement with a diameter of about 5 cm, absence of bleeding during palpation, and no tenderness; a mass was palpable in the right adnexal region, with a smooth surface, poor mobility, and no tenderness. (3) Elevated carbohydrate antigen CA125. (4) The B-mode ultrasound and MRI from the pathological consultation showed HPV-independent invasive adenocarcinoma.

Differential diagnosis was as follows: (1) Cervical cancer. Cervical cancer presents with abnormal vaginal bleeding, which may be accompanied by abdominal pain and other discomforts. Gynecological examination revealed a cauliflower-like change in the cervix that bleeds easily on touch. Imaging revealed cervical space-occupying lesions. Based on the patient’s clinical and examination results, the possibility of the following malignancies is high, and a definitive diagnosis can be made through pathological examination. (2) Benign tumors originating from the ovary. The most common benign tumors originating from the ovary are teratoma, serous cystadenoma, mucinous cystadenoma, or simple cyst. Tumor markers are usually normal, and a definitive diagnosis can be made through pathological examination. (3) Malignant tumors of the ovary or fallopian tube. Malignant tumors of the ovary or fallopian tube typically have a short course and rapid progression, often bilateral, and may present with cachexia. Ultrasound reveals chaotic light clusters and spots within the ovarian fluid-filled area, with unclear boundaries and often abnormally abundant blood flow. There may also be elevated tumor markers such as CA125. Pathological examination can differentiate between these conditions.

### 2.2 Treatment

Preoperative discussion: The patient was diagnosed with cervical adenocarcinoma. The results from pathology suggested HPV-independent invasive adenocarcinoma. This specific type of adenocarcinoma includes subtypes such as gastric-type mucinous adenocarcinoma, clear cell adenocarcinoma, mesonephric adenocarcinoma, and other types of adenocarcinomas. The specific type the patient was diagnosed with is unknown. Following discussions with the pathology department, it was concluded that the patient’s pathological type could not be established due to the limited collection of specimens. Meanwhile, imaging studies revealed uneven thickening of both fallopian tubes with multiple small nodules on the tube wall, and uneven enhancement of both ovaries. Consequently, the potential for adnexal metastasis or even primary adnexal cancer could not be ruled out. Taking all factors into account, surgical intervention was performed.

After excluding surgical contraindications, the patient underwent robotic-assisted laparoscopic extensive hysterectomy, bilateral adnexectomy, greater omentum resection, pelvic and abdominal lymph node dissection, intestinal adhesion release, and cosmetic suture with decorative stitching under general anesthesia.

Intraoperative exploration: The uterus was large with a regular shape, and the cervix was enlarged. The right fallopian tube was tortuous and thickened, adhering to and encapsulating the right ovary, resulting in a cystic mass measuring 6 cm in diameter, and also adhering to and encapsulated by the posterior leaf of the broad ligament. The sigmoid colon adhered to the lateral pelvic wall, and part of the omentum adhered to the posterior wall of the uterus and the pelvic floor peritoneum.

During the operation, the vagina was transected 3 cm below the cervical os, and the entire uterus, bilateral adnexa, part of the greater omentum, and pelvic and abdominal lymph nodes were completely removed. Postoperative prophylaxis against infection and fluid replacement support were administered.

Postoperative pathology: (total uterus and bilateral adnexa, greater omentum, para-aortic lymph nodes, left pelvic lymph nodes, right pelvic lymph nodes) resection specimen: The morphological and immunohistochemical results were consistent with invasive adenocarcinoma of the cervix, which is HPV-independent, and is categorized as a subtype known as gastric-type adenocarcinoma. The cancer tissue had invaded 1/3 of the muscular wall and involved the uterine body, with no intravascular thrombus or neural invasion observed. The bilateral parauterine and vaginal wall resection margins were clear. The endometrium showed proliferative changes. Bilateral fallopian tube tissue was accompanied by a mesosalpinx cyst on the left side. Bilateral ovarian germinal epithelium inclusion cysts were present. (Greater omentum) showed no cancer tissue. No lymph node metastasis (0/19) was observed.

**Figure 4:**
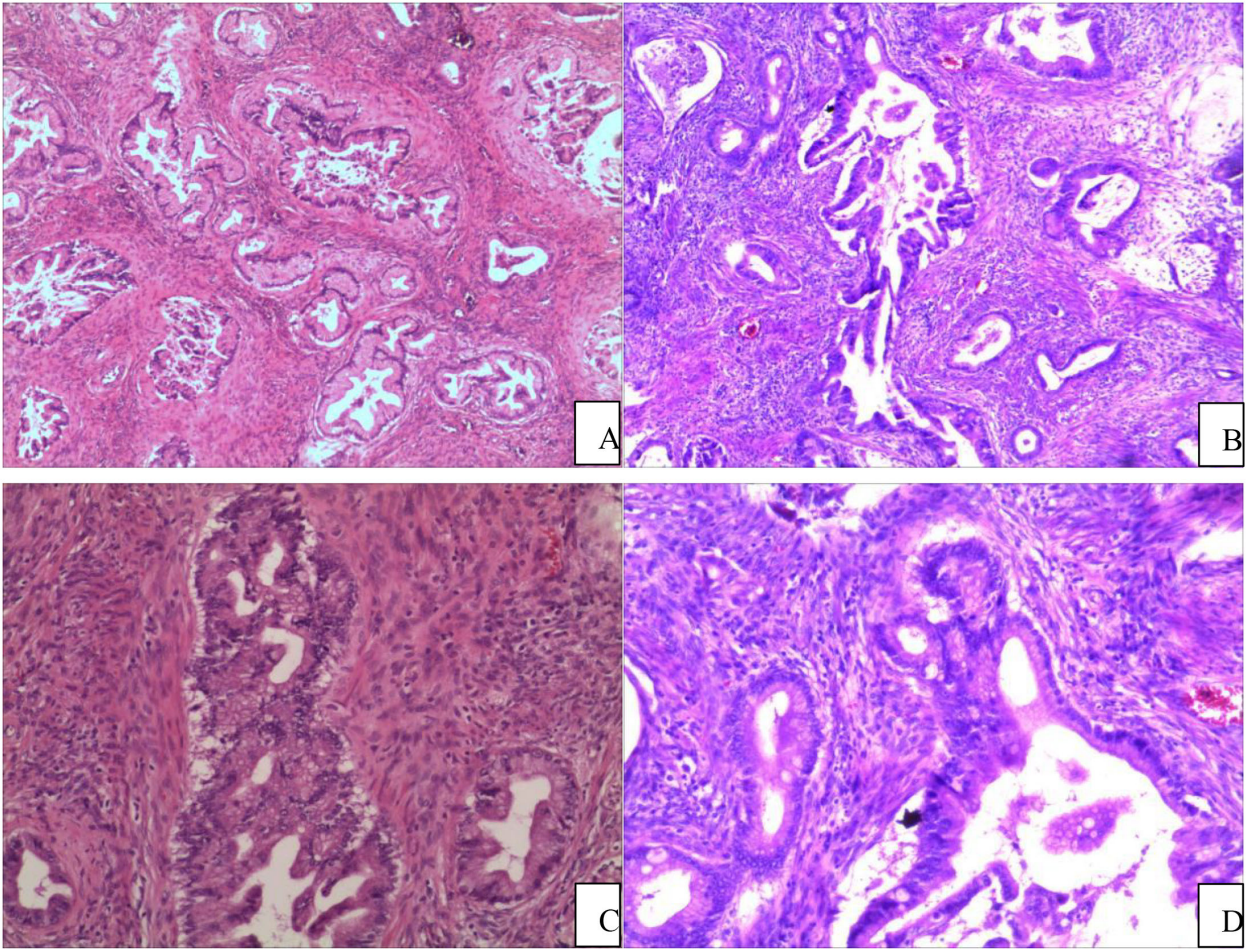
Pathological examination results of cervical biopsy. **(A, B)** Tumor-like proliferative cervical glands exhibit varying sizes, shapes, and disordered distribution. HE×100; **(C, D)**. Glandular cells show minimal pleomorphism, with reactive proliferation of the surrounding stromal tissue and inflammatory cell infiltration. HE×200.

**Figure 5:**
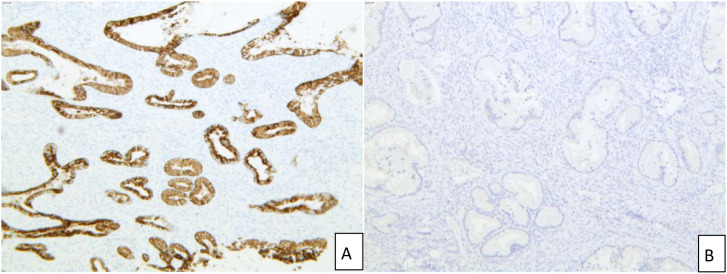
Immunohistochemical results of patients. **(A)** MUC6 shows cytoplasmic positivity, immunohistochemistry ×50; **(B)** P16 shows negativity, immunohistochemistry ×50.

Supplementary postoperative pathology report (**Figure 4**): The neoplastic proliferative cervical glands exhibited varying sizes, shapes, and disordered distribution. The glandular epithelial cells demonstrated high differentiation with minimal cellular pleomorphism. The neoplastic glands have infiltrated the cervical wall to a depth of 2/3. Reactive proliferation of stromal fibrous tissue and inflammatory cell infiltration was observed around the glands. Neoplastic glands were observed to invade blood vessels and lymphatic vessels. The neoplastic glands were rich in neutral mucin. The morphological findings, combined with immunohistochemical results, were consistent with a diagnosis of gastric-type adenocarcinoma of the cervix, exhibiting localized well-differentiated changes. Immunohistochemical results (**Figure 5**) were as follows: CK (+), Vimentin (-), ER auto (-), PR auto (-), P16 (-), Mucin 5AC (+), MUC6 (+), CDX2 (partially +), CK7 (+), CK20 (-), CEA (partially +), Ki-67 auto (40%), CA125 (-).

### 2.3 Treatment outcome, follow-up, and prognosis

Five days after surgery, the patient recovered well without abdominal pain or abdominal distension, and there were no signs of abnormal vaginal bleeding or discharge. Physical examination showed stable vital signs; hence, the patient was discharged. The patient was instructed to follow the discharge instructions, undergo regular follow-up examinations, and seek medical attention if feeling unwell. One month after surgery, a telephone follow-up revealed that the wound had healed well, and the patient reported no abdominal discomfort.

## 3 Discussions

The highly differentiated G-EAC was initially documented by the German gynecologist Gusserow in 1870, who referred to it as malignant adenoma because of its significant aggressiveness. In 1975, Silverbirg and Hurt reclassified it as micro-deviated adenocarcinoma owing to its minimal cellular and structural pleomorphism (8). In 2003, the WHO classification defined it as a micro-deviated type of cervical mucinous adenocarcinoma. In 2014, the World Health Organization (WHO) classification officially recognized G-EAC as a variant of cervical mucinous adenocarcinoma, and MDA was identified as a highly differentiated subtype of G-EAC (9). In the latest 2020 version of the WHO classification, it was classified as cervical HPV-Independent gastric-type adenocarcinoma (10).

The etiology of well-differentiated gastric-endocrine adenocarcinoma (G-EAC is still not fully understood, and it is presently thought to be unrelated to HPV infection. A theory proposing a spectrum of lesion development for G-EAC of the cervix has been supported by various scholars. The proposed progression is as follows: normal cervical mucosa > gastric epithelial metaplasia > cervical lobular hyperplasia > atypical cervical lobular hyperplasia, gastric-type adenocarcinoma *in situ* > well-differentiated G-EAC > poorly differentiated gastric-type adenocarcinoma (11). Furthermore, studies have found that mutations in the tumor suppressor gene *STK11* may be closely related to the occurrence of well-differentiated G-EAC (12). Studies show a close relationship between mutations in the *STK11* tumor suppressor gene and various female reproductive tract cancers, as seen in Peutz-Jeghers syndrome (PJS), which manifests as various female reproductive tract tumors, such as endometrial carcinoma, cervical mucinous adenocarcinoma, and ovarian tubular sex cord tumors.

Highly differentiated G-EAC has a wide range of onset ages, with an average of 42 years. In its early stages, it often presents no symptoms. As the disease progresses, the primary symptoms consist of irregular vaginal bleeding, excessive vaginal secretions, and lower abdominal pain. The disease course is long, ranging from several months to several years. Early gynecological examinations often reveal no abnormalities, and some may show cervical hypertrophy or cervical ulceration. The typical manifestation is a barrel-shaped cervix with hypertrophy, but it is rare. The patient described in this case report presented to the hospital with a chief complaint of vaginal discharge.

The definitive diagnosis of well-differentiated G-EAC requires a detailed histopathological examination of tissue, typically obtained from a biopsy or surgical resection. Purely well-differentiated G-EAC is very rare. Due to the endophytic growth of the lesions, which are often located deep, the challenges in obtaining diagnostic cellular material from these tumors severely limit the utility of cytology alone. Therefore, deep biopsy (>5 mm) or conization is recommended. The pathological characteristics of this disease are summarized as follows (13, 14): (1) Tumor glands are randomly distributed, varying in size and shape, presenting as dendritic or feather-like structures or intraglular papillary protrusions. The glandular epithelial cells and structures show minimal pleomorphism, and mitotic figures are rare. (2) Tumor glands infiltrate the cervical wall to a depth of 2/3 (or >7 mm). Special reactive fibrous stromal tissue proliferation and inflammatory cell infiltration occur around the glands. (3) Tumor glands invade blood vessels, lymphatic vessels, or nerves. They may surround or closely adhere to blood vessels. (4) Tumor glands are rich in neutral mucin, which appears red on PAS staining. (5) Immunohistochemistry shows multiple positive staining for MUC6, Mucin 5AC, HIK1083, and CEA, and multiple negative staining for ER, PR, and CA125. The present case was not tested for HIK1083; however, the other pathological features aligned with earlier literature findings. Due to the lesion’s deep positioning, the presence of minimal cytological and architectural atypia, along with rare mitotic figures, the cervical ThinPrep cytologic test (TCT) came back normal. Furthermore, the initial vaginal biopsy and endometrial curettage only revealed irregular hyperplastic changes.

Imaging examination of well-differentiated G-EAC can reveal tumor size, depth of invasion, surrounding organ involvement, lymph node metastasis, and other conditions, allowing for tumor staging assessment. When imaging well-differentiated G-EAC, MRI with contrast enhancement is a more useful imaging technique for lesion diagnosis and staging than ultrasound and contrast-enhanced CT, and is therefore advised (15). Ultrasound images mainly present as widely distributed cystic or solid masses in the area of the lesion, with the cystic part showing honeycomb-like changes and abundant blood flow signals within the solid lesions (16, 17). Given the non-specific nature of ultrasonographic findings and the challenges in differentiating them from benign lesions, the cervical lesion in this case was initially considered cervical hypertrophy with multiple retention cysts during the initial ultrasound examination. Literature reports indicate that well-differentiated G-EAC typically presents on MRI as multiple cystic or cystic-solid structures of varying sizes located deep within the cervical stroma (6). The MRI presentation in this case demonstrated an enlarged cervix, with diffuse, multiple, poorly defined non-enhancing cystic lesions observed in the cervical region, extending deeply to the subserosal layer. The lesion involved the lower third of the uterine corpus, resulting in similar manifestations in the local uterine wall. These imaging findings are consistent with those reported in the literature. The cystic lesions are considered to result from mucus secretion and retention within the abnormally hyperplastic tumor glands. The extensive invasion of the surrounding pericervical structures and organs signifies the highly aggressive and malignant nature of the lesion. However, these images do not exhibit considerable specificity and lack distinctive features on imaging that differentiate them from other subtypes of gastric-type endocervical adenocarcinoma. Ayumi Ohya et al. investigated the use of the cosmos pattern on MRI to help differentiate gastric-type lesions from other conditions (18). This characteristic sign, however, was absent in the present case, suggesting that the imaging presentations of well-differentiated and poorly differentiated G-EAC may differ slightly. It is also necessary to distinguish the cervical MRI results from lobular endocervical glandular hyperplasia, which is characterized by poorly defined cystic lesions, as opposed to the latter, which typically presents as well-circumscribed lesions, frequently appearing as multiple small cystic lesions or larger cysts encircling a solid nodule. When a small cervical lesion is found alongside extensive surrounding invasion, endometrial and ovarian cancers should be included in the differential diagnosis, with pathological examination being the definitive method for distinguishing them.

The key clinical implication of this report is that the diagnostic evaluation must not be dismissed for patients presenting with persistent abnormal vaginal discharge or bleeding, regardless of a negative HPV test and normal findings from routine cervical screening, since this type of cancer has an insidious onset. Meticulous correlation of clinical, imaging, and pathological features is essential to minimize misdiagnosis and missed diagnosis. A limitation of this study is its failure to propose novel diagnostic or therapeutic approaches.

## 4 Conclusion

Well-differentiated G-EAC is characterized by an insidious onset and rapid progression. Clinically, this disease most commonly presents with non-specific symptoms, primarily abnormal vaginal discharge or bleeding. A definitive diagnosis frequently necessitates tissue obtained through deep biopsy or cervical conization, as cervical screening may produce normal results due to the low sensitivity of cytological examination. Histopathological examination remains the gold standard for confirmation. Imaging studies are crucial for delineating the lesion extent, assessing the depth of stromal invasion, evaluating the involvement of adjacent organs, and detecting lymph node metastasis. All these factors are critical for essential for differential diagnosis, accurate clinical staging, and formulating a suitable treatment plan. Familiarity with the clinical, imaging, and pathological characteristics of this disease can heighten clinical vigilance, potentially reducing the rates of missed diagnosis and misdiagnosis, and thereby preventing delays in optimal therapeutic intervention.

## Data Availability

The original contributions presented in the study are included in the article/Supplementary Material. Further inquiries can be directed to the corresponding author.

## References

[1] Palumbo M Lavitola G Di Filippo C Foreste V Granata M Imperatore O . Impact of Human papillomavirus 9-valent vaccine on viral clearance after surgical treatment:A single-center retrospective observational study. Eur J Obstet Gynecol Reprod Biol. (2025) 310:113994. doi: 10.1016/j.ejogrb.2025.113994, PMID: 40267822

[2] Palumbo M Della Corte L Ronsini C Guerra S Giampaolino P Bifulco G . Surgical treatment for early cervical cancer in the HPV era: state of the art. Healthc (Basel). (2023) 11(22):2942. doi: 10.3390/healthcare11222942, PMID: 37998434 PMC10671714

[3] Xiaodan Z Guangyong C Shoufang H . Interpretation of the classification of uterine body and cervical adenocarcinoma in the 5th edition of the WHO classification of female reproductive tumors. Chin J Pathol. (2021) 50:437–41.

[4] Philip B Hennifer N Robert H . Atlas of Gynecologic Pathology [M]. Translated by Jiang Qingping. Wang Yun. Hu Dan. 4th. Beijing: China Science & Technology Press (2022) p. 147–50.

[5] Loureiro J Oliva E . The spectrum of cervical glandular neoplasia and issues in differential diagnosis. Arch Pathol Lab Med. (2014) 138:453–83. doi: 10.5858/arpa.2012-0493-RA, PMID: 24678677

[6] Yuting B Lei Y Hao Z . Clinicopathological analysis of 27 cases of minimal deviation adenocarcinoma of the cervix. Modern Obstetr Gynecol Prog. (2022) 31:801–6.

[7] Karamurzin YS Kiyokawa T. Parkash V Jotwani AR Patel R Pike MC . Gastric-type endocervical adenocarcinoma:an aggressive tumor with unusual metastatic patterns and poor prognosis. Am J Surg Pathol. (2015) 39(11):1449–57. doi: 10.1097/PAS.0000000000000532, PMID: 26457350 PMC4976691

[8] Mikami Y . Gastric-type mucinous carcinoma of the cervix and its precursors-historical overview. Histopathology. (2020) 76:102–11. doi: 10.1111/his.13993, PMID: 31846534

[9] Kurman RJ Carcangiu ML. Herrington S . WHO classification of tumours of female reproductive organs. 4th. Lyon: IARC (2014) 147–151.

[10] Gynecologic Oncology Group Obstetrics and Gynecology Branch. Chinese Medical Doctor Association . Chinese expert consensus on the clinical diagnosis and treatment of cervical gastric-type adenocarcinoma (2023 edition). Chin J Pract Gynecol Obstetr. (2023) 39:617–25.

[11] Mengfei X Feng Z Xiaoduan C . A case of cervical micro-eccentric adenocarcinoma with poorly differentiated gastric-type adenocarcinoma. Chin J Pathol. (2018) 47:647–8.

[12] Heinritz W Strenge S. Kujat A Hockel M Froster UG . Different phenotypes including gynecological cancer in three female patients with Peutz-Jeghers syndrome and mutations in the STK11 gene. Onkologie. (2008) 31(11):625–8. doi: 10.1159/000162284, PMID: 19145097

[13] Yu H Li Z Yang L Jiang J Bi Y Xiang M . A case report of minimal deviation adenocarcinoma of the cervix and literature review. Int J Gynecol Obstetr. (2018) 45:665–7.

[14] Li H Guo H Han J . Diagnosis and treatment of minimal deviation adenocarcinoma of the cervix. Chin J Cancer. (2008) 30:772–4. 19173810

[15] Stoehr A Nann D. Staebler A Oberlechner E Brucker SY Bachmann C . Difficulties in diagnosis of a minimal deviation adenocarcinoma of uterine cervix diagnosed postoperatively:brief communication and literature review. Arch Gynecol Obstet. (2019) 300(4):1029–43. doi: 10.1007/s00404-019-05286-7, PMID: 31529365

[16] Gong D Li R Tang H Zeng Z Cao C Yang S . One case of gastric-type adenocarcinoma of the cervix [DB/OL. China Clin Case Database. (2023) 5:e00518. doi: 10.3760/cma.j.cmcr.2023.e00518

[17] Yang J Luo X . One case of gastric-type adenocarcinoma of the cervix presenting mainly with vaginal discharge [DB/OL. China Clin Case Database. (2024) 6:E2205. doi: 10.3760/cma.j.cmcr20240828-01735

[18] Ohya A Kobara H Miyamto T Komatsu M Shiozawa T Fujinaga Y . Usefulness of the ‘cosmos pattern’ for differentiating between cervical gastric-type mucin-positive lesions and other benign cervical cystic lesions in magnetic resonance images. J Obstet Gynaecol Res. (2021) 47(2):745–56. doi: 10.1111/jog.14602, PMID: 33331010

